# Perioperative Management for Rectal Migration of a Ventriculoperitoneal Shunt

**DOI:** 10.31486/toj.18.0169

**Published:** 2020

**Authors:** Jeffrey P. Cardinale, Carol O. Carrillo, Jimmie Colón

**Affiliations:** ^1^ Department of Anesthesiology, Ochsner Clinic Foundation, New Orleans, LA; ^2^ The University of Queensland Faculty of Medicine, Ochsner Clinical School, New Orleans, LA

**Keywords:** *Anesthesia*, *hydrocephalus*, *pediatrics*, *surgery*, *ventriculoperitoneal shunt*

## Abstract

**Background:** While ventriculoperitoneal shunt (VPS) is the most commonly performed surgical procedure for treating hydrocephalus, complications following shunt placement are associated with a high mortality rate. Preoperative medical optimization and surgery are the primary means of correcting shunt migration. We present the case of an 11-week-old patient who underwent emergent surgical intervention for transrectal VPS migration and associated infection.

**Case Report:** An 11-week-old female presented with VPS tubing protruding from her rectum. The patient had a history of grade III intraventricular hemorrhage complicated by hydrocephalus status post VPS placement at age 3 weeks. Shunt tap demonstrated gross infection, and she was started prophylactically on broad-spectrum antibiotics. She was taken emergently to the operating room (OR) for VPS externalization and exploratory minilaparotomy. VPS tubing was removed, and the patient was transferred to the pediatric intensive care unit for postoperative management. Cultures confirmed methicillin-resistant *Staphylococcus aureus,* and the patient was treated according to infectious disease recommendations*.* On postoperative day (POD) 5, the patient had a full component VPS replacement. On POD 23, computed tomography scan of the head obtained for lethargy demonstrated a new midline shift, and she was returned to the OR for another VPS replacement. A small abscess was discovered and drained; postoperative cerebrospinal fluid laboratory values normalized after drainage. Once the infectious process cleared, the VPS was internalized on POD 33, and the patient was discharged home on POD 35.

**Conclusion:** Few case reports detail the appropriate anesthetic considerations for cases of VPS migration. This report describes shunt migration pathophysiology and patient assessment with a focus on anesthetic preparation and management for this rare complication.

## INTRODUCTION

Congenital and infantile hydrocephalus occurs in 0.5 to 0.8 per 1,000 live and still births.^1-3^ While medical management can be a temporizing measure for patients showing signs and symptoms of increased intracranial pressure (ICP) secondary to their hydrocephalus (ie, papilledema, bulging fontanelles), surgical drainage is the most effective treatment, and ventriculoperitoneal shunt (VPS) is the most common surgical intervention. Intestinal migration is a rare complication following VPS placement.^4,5^ Surgery is the primary means of correcting VPS migration and typically involves VPS externalization/removal. Preemptive medical and anesthetic management is important for these patients as intraabdominal complications, including unrecognized sepsis and peritonitis, while uncommon, can be devastating.

## CASE REPORT

A 3.13-kg, 11-week-old female presented to the emergency department (ED) with VPS tubing protruding from her rectum and draining into her diaper; radiographs confirmed shunt continuity (Figure). The patient had been born prematurely at 35 weeks and had a history of grade III intraventricular hemorrhage complicated by hydrocephalus status post VPS placement at age 3 weeks. Two 24-gauge peripheral intravenous (IV) catheters were placed upon the patient's arrival to the ED. Shortly thereafter, she experienced a 5-minute tonic-clonic seizure that resolved with IV midazolam; she had no seizure history or prior use of antiepileptic prophylactic medications. Shunt tap demonstrated gross infection with gram-positive cocci in clusters. Laboratory results showed leukocytosis (white blood cells of 36.33 k/μL; reference range, 6-17.5 k/μL) and thrombocytosis (platelet count of 828 k/μL; reference range, 150-350 k/μL), indicating an acute infectious process. The patient also had decreased hemoglobin (6.9 g/dL; reference range, 9-14 g/dL) and hematocrit (22.3%; reference range, 33%-39%). Other laboratory results were within normal limits. She was afebrile with otherwise normal vital signs but was started prophylactically on broad-spectrum antibiotics.

**Figure.**
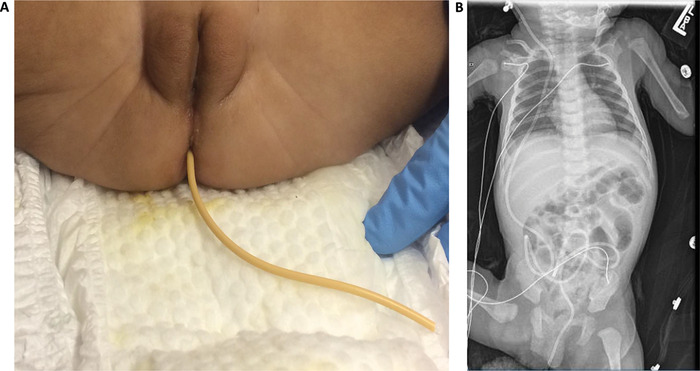
**(A) Transanal ventriculoperitoneal (VP) shunt extrusion from the patient. (B) Radiographic image demonstrating VP shunt continuity.**

Based on her neurologic status, she was taken emergently to the operating room (OR) for VPS externalization and, despite the lack of any outward signs of peritonitis, exploratory minilaparotomy.

Following standard induction and intubation with fentanyl (5 mcg), propofol (7 mg), and rocuronium (1 mg), a right femoral arterial line and a 4-French left internal jugular central line were placed for intraoperative hemodynamic monitoring and for potential medication and resuscitative needs, respectively, as well as for potential postoperative management requirements. She was maintained with sevoflurane. Intraoperatively, the patient received additional doses of rocuronium and fentanyl as needed to maintain ideal operative conditions, she was continued on maintenance fluids, and she remained hemodynamically stable with minimal noted blood loss.

Surgical aspects of the case included removal of the proximal portion of the VPS tubing and reservoir, insertion of a new reservoir, and externalization of the distal portion of the VPS. Neurosurgery administered intrathecal vancomycin (20 mg) and gentamicin (5 mg), and the skin was closed. Simultaneously, through a right lower quadrant minilaparotomy, the surgeons sharply divided the VPS tubing, extracting the proximal portion previously transected in the superior region of the chest by neurosurgery. The distal portion was then removed transanally without difficulty. The area was inspected for hemostasis and continuity of bowel. The surgeons concluded that the patient's bowel had self-sealed and did not require additional direct repair. Incisions were closed. Following skin closure, the patient was reversed with sugammadex (13 mg) secondary to having only 1 of 4 twitches (roughly 2.5 hours total anesthesia time). Her intraoperative arterial blood gas was noteworthy for a hematocrit of 23%; she subsequently received 50 mL (15 mL/kg) of packed red blood cells.

The patient was extubated in the OR and transferred to the pediatric intensive care unit for postoperative management. The initial VPS cultures were positive for methicillin-resistant *Staphylococcus aureus* (MRSA), and she was treated with intraventricular vancomycin and linezolid for 21 days per infectious disease recommendations. On postoperative day (POD) 2, cerebrospinal fluid (CSF) and all subsequent cultures were negative for bacterial growth. On POD 5, the patient had a full component VPS replacement. On POD 23, computed tomography scan of the head obtained for lethargy demonstrated a new midline shift, and she was returned to the OR for another VPS replacement. A small abscess was discovered and drained; postoperative CSF laboratory values normalized after surgical drainage. After confirmation that the infectious process had cleared, the VPS was internalized on POD 33. The patient tolerated oral feeds, gained weight, and was neurologically intact; she was discharged home on POD 35 on antiepileptics (70 mg of levetiracetam twice daily and 16.8 mg of phenobarbital daily). Her posthospital follow-up was otherwise uncomplicated.

## DISCUSSION

Medical management for congenital and infantile hydrocephalus, such as diuretics or serial lumbar punctures, is only a temporizing maneuver.^1^ As such, VPS placement is the most common treatment for obstructive or nonobstructive causes of hydrocephalus in neonates and infants. Complications are common, with an occurrence rate of approximately 40%, and most frequently involve infection, with rates between 5% and 18%.^1-5^ Distal catheter migration, although rare, has been reported at many sites, including the thoracic and abdominal cavities and their occupying organs (lungs, liver, bowel), the intraabdominal wall, bladder, scrotum, vagina, and even the knee.^6,7^ Spontaneous bowel perforation is rare (0.01% to 0.7% of VPS complications) but carries a high mortality rate of approximately 15% secondary to intraabdominal and/or intracranial infections.^1-7^ Interestingly, >40% of abdominal migrations are asymptomatic.^8^

The etiology of VPS bowel perforation is largely unknown. Potential causative factors include longer catheter length, a sharp/heavy catheter tip, fibrous adhesions, and chronic inflammatory processes from steady pressure that causes boring of the catheter through the visceral wall.^6,9^ In children specifically, weaker intestinal musculature and stronger peristaltic activity may contribute to increased complication rates.^9^ While surgery is the definitive management for VPS migration, the need for laparotomy continues to be debated.^2,6,8^ The scope of surgical intervention depends upon where the catheter migrated and the presence of sepsis, peritonitis, or abscess formation, lending increased importance to preoperative management and careful diagnosis. Clear signs of peritonitis can be difficult to distinguish and may not always be present.^5,8^ Our patient did not display a clear-cut intraabdominal process. Her cultures yielded MRSA vs the more likely gram-negative source given the VPS catheter migration through the gastrointestinal tract, but secondary to her meningitis, the surgical teams felt strongly about proceeding with a minilaparotomy for confirmation and precise catheter extraction.

From an anesthetic perspective, case preparation involves consideration of any potential outcome, including sepsis and intraabdominal peritonitis. Long-term postoperative concerns should also be considered, including vascular access and hemodynamic monitoring, and invasive lines may be indicated for optimal perioperative management. Anesthetic induction, maintenance, and emergence are based on patient condition, and in this case, we followed standard pediatric procedures.

Elevated ICP can occur in patients with hydrocephalus and VPS, although the occurrence is less likely if shunt drainage remains patent or signs of increased ICP (ie, lethargy, poor feeding, bulging fontanelles) are absent. Induction agents, including propofol, can decrease ICP secondary to decreased cerebral blood flow (CBF). Of the inhalational anesthetics, sevoflurane produces the mildest increase in CBF. Slight hyperventilation may be helpful in decreasing CBF. Other considerations include maintenance of normothermia, adequate fluid management, and perioperative pain management. While this case was without incident, preparation for the unexpected was warranted.

## CONCLUSION

Distal VPS migration is a rare complication with potentially devastating consequences. While many of these cases have positive outcomes, the high mortality rate associated with shunt migrations hints at the seriousness of these occurrences. While the patient's initial surgery and anesthetic course were without complication, these cases demand a need for a proactive medical, surgical, and anesthetic approach given the potential level of uncertainty regarding the scope of intraabdominal involvement and septic risks. Adequate preparation for unexpected events is important in providing effective and safe anesthetic for VPS surgical management.

## Notes

This article meets the Accreditation Council for Graduate Medical Education and the American Board of Medical Specialties Maintenance of Certification competencies for Patient Care and Medical Knowledge.
